# Optimization of hydroxyurea in sickle cell disease in Brazil

**DOI:** 10.1016/j.htct.2025.103826

**Published:** 2025-05-01

**Authors:** Clarisse Lobo, Ana Cristina Silva-Pinto, Rodolfo Delfini Cançado

**Affiliations:** a Instituto Estadual de Hematologia do Rio de Janeiro HEMORIO, Rio de Janeiro, RJ, Brazil; b Faculdade de Ciências médicas da Santa Casa de São Paulo, and Samaritano Hospital - São Paulo, São Paulo, Brazil; c Faculdade de Ciências Médicas da Santa Casa de Sao Paulo, Sao Paulo, Brazil

**Keywords:** Sickle cell disease, Hydroxyurea therapy, Innovative drug development, Prognosis

## Abstract

Despite sickle cell disease (SCD) being a well-recognized and highly prevalent condition identified early through neonatal screening programs, it represents a substantial public health challenge due to high morbidity and premature mortality rates. Hydroxyurea (HU) is the only available disease-modifying therapy for SCD approved in Brazil. Indeed, its underutilization highlights the need for improved therapeutic strategies to enhance adherence and management of SCD. Innovative formulations of HU might favor treatment adherence and precise dosing. Thus, we aimed to describe HU's pharmacological characteristics, clinical efficacy, and tolerability, including dose escalation. Recent interventional and observational studies revealed the efficacy and safety of an innovative formulation: dispersible scored tablets of 100 mg and 1000 mg, allowing easier dose adjustments and, consequently, more precise dosing. The 100 mg tablets scored can be cut into two parts of 50 mg, and the 1000 mg tablets can be cut into four parts of 250 mg. The fractionating dose is possible due to the formulation technology that allows the tablet to be cut with a uniform amount of drug in each part. This new formulation of HU, suitable for children, may influence the prognosis of SDC, regardless of associated symptoms.

## Introduction

Hemoglobinopathies, including sickle cell disease (SCD), are the most common inherited disorders worldwide.^1^ Sickle cell disease (SCD) is a term that encompasses a variety of genetic disorders characterized by the presence of abnormal hemoglobin, primarily hemoglobin S (HbS).^2^^,^^3^ The most prevalent genotype are: HbSS usually called sickle cell anemia (SCA), HbSC, and HbSβ-thalassemia subtypes characterized by mutations in the gene encoding the hemoglobin subunit β (HBB).^2^^,^^3^ The most common form of SCD is sickle cell anemia (SCA), which occurs when an individual inherits two copies of the HbS gene (HbSS). In contrast, sickle cell disease with hemoglobin C (HbSC) occurs when an individual inherits one HbS gene and one hemoglobin C gene (HbC). The clinical manifestations of SCD can vary significantly between these genotypes, with SCA generally presenting more severe symptoms and complications compared to HbSC disease.^4^

The burden of SCD particularly affects low-middle income countries (LMICs) from sub-Saharan Africa, Arab countries, the Mediterranean, the Indian subcontinent, the Caribbean, and South America, as well as African Americans and descendants of immigrants from these countries all over the world.^5^^,^^6^ One of the highest incidences of SCD, however, is reported in Africa with an estimated global birth rate of 300,000 annually^6^. There are an estimated 90,000 to 100,000 individuals with SCD in the USA and 60,000 to 100,000 individuals living with SCD in Brazil.^6^^,^^7^ Of note, SCA, the most common genotype and most severe variant of SCD, makes up >80% of all SCD cases, showing its dominance among sickle cell disorders.^8^ Regarding mortality, significant death rates presents an alarming situation occur in LMICs where SCD is more prevalent.^9^ SCA in children is an alarming situation due to high morbidity and mortality from vaso-occlusive crises, acute chest syndrome, and infections.^10^, ^11^, ^12^ In Brazil, one of the countries most affected by the disease, Lobo et al. reported in a large sample of 1676 patients with SCD from a referral tertiary hospital (1998 to 2012), 281 deaths (mortality rate: 16.8%).^13^ The most frequent causes of death were infection, SCA, overt stroke, organ damage, and sudden death during painful crises.^13^ Populational-based data revealed 6553,132 deaths in Brazil from 2015 to 2019, identifying 3320 deaths from SCD (mean age: 32 years).^14^ Also, an estimated annual economic burden of approximately 400 million USD was estimated from a reference center in Brazil.^15^

Despite SCD being a well-recognized and highly prevalent condition identified early through neonatal screening programs, it represents a substantial public health challenge due to high morbidity and premature mortality rates. Hydroxyurea (HU) is the only available disease-modifying therapy for SCD approved worldwide. Innovative formulations of HU as dispersible scored tablets might favor treatment adherence and precise dosification. Our objective was to detail an overview of pharmacological characteristics, clinical efficacy, safety profile, and current guidelines regarding the use of hydroxyurea in the management of SCD in Brazil.^16^^,^^17^ Indeed, we update SCD treatment focused on HU and its innovative formulation in dispersible scored tablets. Treatment of SCD

The management of SCD patients may include using hydroxyurea (HU), folic acid, blood transfusion, iron chelation, antibiotic therapies, vaccination, hematopoietic stem cell transplantation (HSCT) and gene therapy.^1^^,^^2^^,^^18^

### Hydroxyurea

HU is an inhibitor of ribonucleotide reductase with many beneficial effects for treating people with SCD, including increasing HbF concentration in red blood cells (RBC), improving nitric oxide metabolism, reducing red cell-endothelial interaction, and erythrocyte density.^1^^,^^2^^,^^25^ Such disease-modifying effects have been shown to decrease vaso-occlusive crises (VOC), SCA, the number/length of hospitalizations, and the need for transfusions, noticeably reducing the mortality rate and improving overall survival.^1^^,^^2^^,^^18^

HU has a direct effect on the pathophysiological mechanisms of SCD, acting not only by increasing the synthesis of HbF but also by promoting a decrease in the number of neutrophils and erythrocyte adhesion molecules, thus directly contributing to the reduction of inflammatory phenomena and vaso-occlusion.^1^^,^^2^ It was also observed that HU therapy is associated with an increase in intravascular and intraerythrocytic production of nitric oxide facilitating vasodilation, which represents another direct effect of the drug on the pathophysiological mechanisms of SCD.^1^^,^^2^^,^^25^^,^^26^

In clinical practice, the beneficial effects of HU are observed in the first weeks after its introduction.

The main benefits of HU scientifically proven are the elevation of HbF to a desirable level of 20% or more, with consequent prevention of sickling and a significant reduction in disease complications (morbidity) and, almost 50% in mortality.^19^ Data shows that this result is usually achieved in patients who received escalating doses to a maximal tolerated dose (MTD).^20^ In addition, it is cost-effective based on fewer hospitalizations, and positive impact on quality of life, physical and psychosocial, with oral formulation, which favors a better adherence to treatment.^21^, ^22^, ^23^, ^24^

HU is still the most used disease-modifying therapy approved for SCD worldwide. Since 1996, HU has been released in the United States and Europe to treat SCA (the severe genotypes with more prominent anemia, hemolysis, and clinical complications). Of note, patients with "milder" genotypes (e.g., HbSC) were excluded from randomized clinical trials (RCTs) that were used to support the Food and Drugs Administration (FDA) approved. Due to the benefits of HU observed in adults, this drug is currently under approval for children older than 9 months, particularly SCD with severe manifestations of the disease in the United States and the United Kingdom.^25^, ^26^, ^27^

In Brazil, HU has been recommended for adults and children (> 2 years old) with a worse prognosis since 2002. In 2018, the Brazilian treatment protocol of SCD, “Protocolo Clinico e Diretrizes Terapêuticas (PCDT) de Anemia Falciforme” included using HU for children from 9 months of age considering situations of chronic organ damage and hemolysis as indications for drug use.^28^ The inclusion of patients with SCD and 9 months old to treatment of HU should consider the following additional criteria: dactylitis (in the first year of life); Hb concentration lower than 7 g/dL (average of 3 values outside of an acute event); or leukocyte count higher than 20,000/mm^3^ (average of 3 values outside of an acute event).^28^ In 2024, the ”Consensus of the Brazilian Association of Hematology, Hemotherapy and Cellular Therapy (ABHH) and the Brazilian Ministry of Health” included HU treatment of children under two-years in the presence of HbSS, HbSB^0^, HbSD Punjab and HbSB+ with <10% of Hb A, regardless any symptoms.^29^ In 2024, the Brazilian treatment protocol of SCD, “Protocolo Clinico (PCDT) de Anemia Falciforme” was updated, according to these same criteria.

The Therapeutic Response Evaluation and Adherence Trial (TREAT) has shown that early initiation of HU, with personalized, pharmacokinetically guided dosing to optimize benefits and reduce toxicity, has the potential to be nearly curative. This approach leads to high levels and pancellular expression of Fetal hemoglobin (HbF) within red blood cells, resulting in a significant reduction of clinical complications in most patients who adhere to the treatment.^30^

### Pre-treatment considerations

Before initiating the treatment, it is recommended the following tests:

A complete blood count (CBC) that includes mean corpuscular volume (MCV), white blood cell (WBC) differential, reticulocyte count, and platelet coun*t; h*emoglobin (Hb) electrophoresis with HbF measurement (high-performance liquid chromatography HPLC, if available); a comprehensive metabolic profile that includes renal and liver function tests, and a pregnancy test for women of reproductive age. It is essential to highlight that even in patients with baseline elevation of HbF, it should not affect the decision to initiate HU therapy, and both males and females of reproductive age should be counseled regarding the need for contraception being HU a cytostatic drug which a potential teratogenic concerns.^1^^,^^2^^,^^25^^,^^26^^,^^28^^,^^29^

### Initial dosing

According to international guidelines^27^^.^^23^^,^^24^^,^ including the Brazilian^28^^,^^29^, HU must be used orally at 15 to 35 mg/kg daily. For adults, the initial dose is 15 mg/kg/day, and 20 mg/kg/day for infants and children. The HU dose should be reduced to 5–10 mg/kg/day for adult patients with chronic kidney disease.^27^, ^28^, ^29^

Regarding its pharmacokinetics, HU is rapidly absorbed after oral administration, reaching a maximum plasma level between 20 and 30 min (fast absorbers) and 60 min (slow absorbers), with a ½ time life of three to four hours. HU is metabolized in the liver and excreted via renal (80%).25^,^26With an initial once-daily dose for adults of 15 mg/kg/day, or children 20 mg/kg/day monitoring the number of leukocytes and platelets (CBC) every two weeks is recommended. 25 This initial dose should be increased by 5 mg/kg/day every 8 to 12 weeks as titration, with the goal being to reach the maximum tolerated dose (MTD). 25 Based on the published data, the MTD is the highest dose capable of promoting the most prominent improvement in the clinical and laboratory parameters, without the occurrence of hematological, hepatic (defined as an increase of twice the maximum reference value of transaminases), renal (increased urea and creatinine) or gastrointestinal toxicity. MTD should not exceed 35 mg/kg/day.^25^^,^^26^

### Monitoring, dosage modification and response

It is advisable to monitor CBC with WBC differential and reticulocyte count every 4 weeks when adjusting dosage. The target absolute neutrophil count is ≥2000/μL. However, younger patients with lower baseline counts may safely tolerate counts down to 1250/μL and platelet counts ≥80,000/μL.^1^^,^^2^^,^^18^^,^^25^^,^^26^^,^^27^^,^^28^^,^^29^

If neutropenia or thrombocytopenia occurs, HU dosing should be suspended, and monitor CBC with WBC differential weekly. When blood counts recover, restart HU at 5 mg/kg/day lower than the previous dose.^1^^,^^2^^,^^18^^,^^25^^,^^26^^,^^28^^,^^29^

If escalation is warranted based on clinical and laboratory findings, increase by 5 mg/kg/day every 8 weeks until mild myelosuppression (absolute neutrophil count 2000/μL to 4000/μL) is achieved up to a maximum of 35 mg/kg/day.^1^^,^^2^^,^^18^^,^^25^^,^^26^^,^^28^

After a stable dose is determined, follow-up involves conducting blood counts with MCV, WBC differential, reticulocyte, platelet counts, and liver transaminase levels every 2–3 months. The HbF level should be checked every six months; however, its increase can vary significantly and does not always correlate with the clinical response. ^1^^,^^2^^,^^18^^,^^25^^,^^26^^,^^28^^,^^29^

Monitor Hb, MCV, and HbF levels for evidence of consistent or progressive laboratory response. A clinical response to treatment with HU may take 3–6 months. Therefore, a 6-month trial on the MTD is required before considering discontinuation due to treatment failure, whether due to lack of adherence or failure to respond to therapy. ^1^^,^^2^^,^^18^^,^^25^^,^^26^^,^^28^^,^^29^

A lack of increased MCV and/or HbF does not indicate discontinuing therapy. Hydroxyurea therapy should be continued during hospitalizations or illness. Patients should be constantly reminded that the effectiveness of HU depends on their adherence to daily dosing.^1^^,^^2^^,^^18^^,^^25^^,^^26^^,^^28^^,^^29^

### Impact of HU on clinical outcome and mortality

Hydroxyurea has demonstrated effectiveness in reducing mortality and acute events in patients with HbSS. The Multicenter Study of Hydroxyurea Use found a 40% reduction in mortality among adults with HbSS and HbF >5.0 g/L treated with HU compared to those untreated.^26^ In a 17-year prospective non-randomized study, HU therapy reduced mortality by 73%.^31^ Similar results were seen in a Brazilian retrospective trial of children with SCD (ages 3–18).^32^ In this study among 1760 patients, 267 received HU at an average dose of 20.8 mg/kg/day during a mean follow-up of 2 years. HU significantly increased Hb, HbF, and MCV levels while reducing leukocytes, neutrophils, platelets, and reticulocytes. The HU group had a 50% reduction in hospital admissions (0.4 to 0.2 events/year), a 60% decrease in hospital stay length (4 to 1.6 days), and a 35% reduction in emergency room visits. Survival was significantly higher in the HU group (99.5% vs. 94%, *p* = 0.01), with an 87% reduction in mortality risk (OR 0.13, 95% CI 0.02–0.99, *p* = 0.049). Adverse effects were mild; no severe neutropenia or thrombocytopenia was observed.^32^ Another Brazilian single-center study showed a significant reduction in infectious episodes with HU treatment (1.03 to 0.5, *p* = 0.047).^33^ The Baby Hug study, a multicenter randomized clinical trial performed in 13 centers in USA, included children aged 9–18 months with HbSS or Sβ0 thalassemia, randomized to 20 mg/kg/day of liquid HU (*n* = 83) or placebo (*n* = 84) for 2 years. Of note, the study sample was not selected for clinical severity. As the main findings, HU compared to placebo significantly reduced pain episodes (177 vs. 375, *p* = 0.02) and dactylitis (24 vs. 123, *p* < 0.0001) and showed trends toward lower rates of acute chest syndrome (ACS), hospitalization and transfusions. HU also increased Hb and HbF and lowered leukocyte counts. Toxicity was mild, mostly limited to moderate neutropenia.^34^ The TWiTCH (Transfusions Changing to Hydroxyurea) was a non-inferiority, multicenter, open-label, phase 3 study, purposed to evaluate whether Hydroxyurea (HU) could serve as an effective alternative to chronic red blood cell (pRBC) transfusions, which are the standard treatment, in preventing primary strokes in children with sickle cell anemia. The study involved 121 children aged 4 to 16 years, who had to have a transcranial Doppler (TCD) reading of ≥ 200 cm/s, have received pRBC transfusions for at least one year, and did not exhibit severe vasculopathy on magnetic resonance angiography (MRA). The participants were randomly assigned to two groups: one group continued receiving packed red blood cell (pRBC) therapy, which included 61 children, while the other group switched to hydroxyurea (HU) treatment at MTD, comprising 60 children. In the group that continued pRBC therapy, the average TCD velocity was 145 ± 21 cm/s, while in the group that switched to hydroxyurea (HU), it was 145 ± 26 cm/s. Children in the pRBC group maintained HbS levels below 30% and had an average HbF level of 25%, which was similar to the children in the HU group. The study was halted during the initial analysis after it was found that HU was not inferior to pRBC transfusions in preventing strokes. The maximum mean TCD velocity in the HU group was 143 ± 1.6 cm/s, compared to 138 cm/s in the pRBC group (95% confidence interval, 95% CI: 4.54, 0.10 – 8.98; non-inferiority *p* = 8.82 × 10^−16).^35^ Additionally, a trial in sub-Saharan Africa comparing fixed doses of HU (20 mg/kg/day) with the MTD (up to 30 mg/kg/day) demonstrated a 79% reduction in hospitalizations, a 70% decrease in transfusion rates, and reductions in acute chest syndrome (73%) and vaso-occlusive crises (57%), with similar toxicity across both groups.^36^

## Hydroxyurea precision dosing

The HU formulation for SCD (Sickle Cell Disease) is available in both tablet and capsule forms, marketed under the trade names Siklos® and Tepev FF®. In Brazil and many other countries, only 500 mg capsules have been available, under off-label use, for adults and children in the recent past. However, when adjustments other than 500 mg or its multiples are necessary, the capsule formulation can pose several challenges. These include the need to manipulate the medication at home, the potential waste of leftover doses, and an increased risk of dosing errors due to the complexity of the dosing and dilution process.^1^^,^^2^^,^^23^

Particularly, the recent formulation of HU (Siklos®) is an innovative drug in water-dispersible scored tablets of 100 mg and 1000 mg, which allows dose adjustments for easier understanding of directions for patients or caregivers, thus favoring treatment adherence and precise dosification. The 100 mg scored tablets can be cut into two 50 mg parts, and the 1000 mg scored tablets can be cut into four parts of 250 mg each. The fractionating dose is possible due to the formulation technology that allows the tablet to be cut with a uniform amount of drug in each part.^37^^,^^38^

The European Sickle Cell Disease Cohort – Hydroxyurea (ESCORT-HU study) is a, prospective cohort multicenter study conducted in SCD patients treated with HU according to current clinical practice in Europe. Siklos® was administered to children and adults. The patients previously treated with the capsule formulation (500 mg) were switched to HU tablets and included in the study as non-naive patients. The other half of the patients enrolled in the ESCORT-HU started their HU treatment with the tablets (never users, naïve patients). Over 1906 participants aged 2 years and older (55% adults) with symptomatic SCD were enrolled, with a median follow-up of 45 months covering 7309 patient-years of observation. HU average doses were 20.6 mg/kg/day for children and 16.3 mg/kg/day for adults. statistically significant reductions were observed in VOC episodes lasting >48 h, acute chest syndrome, hospitalizations and blood transfusion rates within the first 12 months compared to the previous year.^38^ The most common adverse effects were transient neutropenia and thrombocytopenia, with no new toxicity reported as shown in Table 1.

**Table 1:** Clinical and laboratory parameters of efficacy one year before and after the treatment of HU fractionable tablets in 1903 participants from the ESCORT-HU study according to age group.

	Age <18 years (*n* = 849)					Age >18 years (*n* = 1054)			
Period of HU fractionable treatment	Number	Previous year	After 1 year*	Change Pre-post (p value)		Number	Previousyear	After1 year*	ChangePre-post(p value)
N^o^. of VOC, mean (±SD)	682	1.6 (2.1)	0.9 (1.6)	−50% (<0.05)		907	1.8 (2.6)	0.9 (1.9)	−38% (<0.05)
N^o^. of ACS, mean (±SD)	708	0.3 (0.7)	0.1 (0.3)	−67% (<0.05)		940	0.3 (0.6)	0.1 (0.4)	−67% (<0.05)
N^o^ of hospitalizations, mean (±SD)	681	1.7 (1.8)	0.93 (1.5)	−46% (<0.05)		930	1.3 (1.8)	0.7 (1.3)	−44% (<0.05)
Days of Hospitalization due to SCD, mean (±SD)	628	9.7 (12.1)	5.5 (10.1)	−46% (<0.05)		833	8.1 (13.4)	4.4 (10.8)	−43% (< 0.05)
No. of patients (%) with at least one blood transfusion	810	369 (45.6)	199 (24.6)	−21% (<0.001)		1024	400 (39.1)	177 (17.3)	−21.8% (<0.001)

Abbreviations: ACS, acute chest syndrome; HU, hydroxyurea; SCD, sickle cell disease; VOC, vaso-occlusive crises.

Adapted from Montalembert et al.38.

*After 1-year: Within 1-year after the HU fractionable tablet.

The subgroup of patients receiving HU treatment (*N* = 926) who transitioned to the dose-adjusted tablet (HU non-naïve) were compared to those for whom HU tablets were introduced during the ESCORT-HU study (HU naïve) (*N* = 976). In the subgroup of 926 patients, there was an increase in MCV from 86.15±14.37 to 94.72±16.39 after 12 months of Siklos use, which suggests an increase in adherence since these patients were already using HU (non-adjustable dose of 500 mg) in the previous year, it was also possible to observe in this group, a decrease in the number of VOC events from 2.68 to 2.89 (95%CI) to 0.68–0.86 (95%CI), a decrease in the number of acute chest syndrome (ASC) 0.85- 1.54 (95%CI) to 0.08–0.13 (95%CI) and in the number of hospitalizations from 2.27 to 2.44 (95%CI) to 0.64–0.81(95%CI). There was no increase in adverse events compared to HU non-naïve with HU naïve patients. These data favored medication adherence with the Siklos® adjusted daily dose, however, no specific analysis was performed ^37^. The ESCORT-HU trial provides evidence about long term benefit and safety in a large group of participants enrolled. The clinical and laboratory outcomes with HU adjusted tablet (precision dosing: fractionable tablet) are shown in Table 2, Table 3.^37^

**Table 2:** Number of clinical outcomes before and after one year of treatment with HU fractionable tablets in the subgroup of participants from the ESCORT-HU.

Previous treatment with another HU formulation	HU Non-naïve (*N* = 926)		HU Naïve (*N* = 976)	
	Previous year	After 1 year*	Previous year	After 1 year*
N^o^ of VOC, Mean ± SD (95% CI)	2.79 ± 2.66 (2.68 to 2.89)	0.77 ± 1.37 (0.68 to 0.86)	2.96 ± 2.69 (2.79 to 3.12)	0.79 ± 1.94 (0.66 to 0.92)
N^o^ of ACS, Mean ± SD (95% CI)	1.20 ± 0.53 (0.85 to 1.54)	0.11 ± 0.38 (0.08 to 0.13)	1.30 ± 0.81 (0.79 to 1.80)	0.07 ± 0.27 (0.05 to 0.09)
N^o^ of hospitalizations, Mean ± SD (95% CI)	2.36 ± 1.85 (2.27 to 2.44)	0.73 ± 1.26 (0.64 to 0.81)	2.51 ± 1.63 (2.40 to 2.61)	0.67 ± 1.33 (0.58 to 0.75)

Abbreviations: ACS, acute chest syndrome; HU, hydroxyurea; SCD, sickle cell disease; VOC, vaso-occlusive crises; 95%CI, 95% confidence interval.

Adapted from Galactéros et al., 2022.37.

*After 1-year: Within 1-year after the HU fractionable tablet.

**Table 3:** Biological parameters before and after 12 and 24 months of the treatment with HU fractionable tablets in the subgroup of participants from the ESCORT-HU.

Previous treatment with another HU formulation		HU Non-naïve (*N* = 926)	HU Naïve (*N* = 976)
Hb (g/dl), mean ± SD	Baseline 12 months 24 months	9.01 ± 1.47 9.01 ± 1.56 9.04 ± 1.48	8.63 ± 1,60 9.01 ± 1.41 9.01 ± 1.46
HbF (g/dl), mean ± SD	Baseline 12 months 24 months	13.58 ± 9.44 16.24 ± 10.08 16.20 ± 9.80	7.12 ± 5.94 15.05 ± 9.67 15.34 ± 19.15
MCV (fl), mean ± SD	Baseline 12 months 24 months	86.15 ± 14.37 94.72 ± 16.39 95.31 ± 15.05	85.07 ± 12.34 89.55 ± 13.44 89.45 ± 12.91
Neutrophils (10^9^/L), mean ± SD	Baseline 12 months 24 months	4.78 ± 2.44 4.41 ± 2.51 4.07 ± 2.01	5.54 ± 3.06 4.61 ± 2.98 4.23 ± 2.49

Adapted from Galactéros et al.37.

### HU adverse effects and toxicity

Overall, the chronic use of HU is not related to severe adverse effects (AEs) such as death in the context of SCD, mainly SCA.^25^^,^^34^^,^^37^^,^^38^^,^^39^ Common AEs reported by most studies (RCTs and observational) performed in adults and children with SCD included hematological symptoms (myelosuppression), gastrointestinal issues (nausea, diarrhea, constipation, anorexia), vasculitis toxicities, macrocytosis, onychomadesis, rash, hair loss, headache, dizziness, stomach pain, swelling, dry skin and nail pigmentation.^25^^,^^34^^,^^37^^,^^38^^,^^39^ Neutropenia and thrombocytopenia, the most common effects observed with HU treatment, were reversed with temporary drug interruption, usually recovered within two weeks.^25^^,^^34^^,^^37^^,^^38^^,^^39^ Particularly, the Baby-HUG trial (2011) performed in children with SCA, gastroenteritis and dactylitis occurred less frequently in those receiving hydroxyurea compared to placebo (*p* < 0.001). Other less frequent AEs, sepsis or bacteraemia occurred three times in those receiving HU and six times in the placebo group, but without statistical significance. Episodes of splenic sequestration were also equal in the two groups. Toxicity was limited to mild-to-moderate neutropenia (500–1250/mm^3^), higher in the HU group than placebo.^34^ In the RTC of Charache et al.^25^ performed in adults with SCA, AEs were equally common in both placebo and active treatment groups. In the ESCORT-HU, real-world observational cohort performed with HU fractionable (scored/breakable) tablets, no reported differences between non-users (naïve) and previous users of HU capsules (non-naïve) regarding most common AEs incidence (<5% in both groups) were neutropenia, thrombocytopenia and dry skin.^37^

Other toxicities reported such as renal and hepatic toxicities are rare, and there is no evidence of increased cancer incidence with prolonged HU use. In males treated during childhood, no toxic effects on sperm tests were found, and spermatogenesis toxicity is not a significant concern in boys requiring HU treatment before puberty.^1^^,^^2^^,^^37^^,^^38^^,^^39^

### Barriers to the use of HU

Lobo et al., analyzed 1144 patients with SCD at HEMORIO (Rio de Janeiro), and observed that HU was prescribed to 40.5% of the children and 36.4% of adults.^32^ Carneiro-Proietti et al. reported using HU in 29.3% of children (458 of 1104 patients) and 36.3% of adults (447 of 1044 patients).^40^

The attainment of optimal HU benefits requires selecting and maintaining the proper dose, which varies widely from one patient to the next. Inadequate HU dosing results in suboptimal clinical responses, poor medication adherence, and decreased utilization of HU as a disease-modifying and life-saving drug.^41^

HU is underutilized partly due to a lack of awareness of its benefits on the part of patients and providers, which compromises patient adherence; that is the primary reason why HU therapy is ineffective in children and adults with SCD, others reasons are: concerns regarding adverse events (i.e., myelosuppression), need for regular laboratory monitoring, uncertainties surrounding possible adverse effects on reproduction and fertility.^1^^,^^2^^,^^39^^,^^42^

Moreover, the new tablet formulation of 100 mg and 1000 mg scored tablets enables physicians to prescribe an accurate dose, as well as to perform dose escalation in 50 mg or 250 mg increments progressively and continuously until reaching the maximum tolerated dose. This approach is recommended and aligns with the pharmacodynamic features of hydroxyurea.^43^

To optimize HU treatment for both children and adults, it is crucial to address barriers related to its use, ensuring treatment is tailored to the patient's body weight and biological and clinical response. The introduction of fractionable (scored/breakable) tablet formulations, especially for children under 6–7 years of age, will facilitate appropriate dosing and may improve adherence.^37^^,^^38^ This is because tablets allow for more precise dose adjustment, which is particularly important for patients who require individualized titration. The ability to tailor the dosage to each patient's specific needs enhances the effectiveness of the treatment and reduces the risk of under or over-dosing. Moreover, the convenience and ease of administering tablets including the benefits of dissolving water if needed may contribute to better treatment adherence. Patients are more likely to consistently take their medication when the process is straightforward and manageable, especially in the context of chronic conditions like SCD, where long-term adherence is critical. Improved adherence, in turn, positively impacts clinical outcomes, as patients are more likely to experience the full therapeutic benefits of the treatment. Therefore, the results of clinical studies suggest that tablet formulation can be a key factor in enhancing adherence, in the observed therapeutic success. Additional studies about the adherence of HU in fractionable (scored/breakable) tablets are needed since no specific evaluation was performed in the ESCORT-HU trial.^37^^,^^38^

## Conclusion

Hydroxyurea (HU) has significantly altered the natural history of SCD globally, reducing mortality rates and improving overall survival. Despite its cost-effectiveness, efficacy and safety, HU should not be overshadowed by new SCD treatments. In Brazil, although HU is available through the Unified Health System (SUS), it is often administered in suboptimal doses with poor adherence, leading to high mortality rates, particularly among children. The introduction of new technologies for precise dosing and improved adherence is timely. However, ongoing efforts are needed to raise awareness among prescribers, patients, and parents, and to ensure greater commitment from governments, manufacturers, and society to guarantee HU's availability and accessibility for all SCD patients.

## Conflicts of interest

**CL** is a consultant from EMS, Masters Speciality Pharma, Global Blood Therapeutics (GBT), Agios, Pfizer, and Novartis. ORCID: 0000–0002–4262–5792, **ACSP** is a consultant from EMS, Masters Speciality Pharma, Global Blood Therapeutics (GBT), Chiesi Farmacêutica S.p.A., and Novartis. ORCID: 0000–0002–41,042,296, **RDC** is a consultant from Pfizer, CSL Vifor, Masters Speciality Pharma, Global Blood Therapeutics (GBT), and Novartis. ORCID: 0000–0003–32,693,780
